# University student-led public engagement event: increasing audience diversity and impact in a non-science space

**DOI:** 10.1099/acmi.0.000534.v3

**Published:** 2023-08-29

**Authors:** Melissa M. Lacey, Kelly Capper-Parkin, Rachel Schwartz-Narbonne, Kate Hargreaves, Catherine Higham, Catherine Duckett, Sarah Forbes, Katherine Rawlinson

**Affiliations:** ^1^​ Biomolecular Sciences Research Centre, Department of Biosciences and Chemistry, Sheffield Hallam University, Sheffield, UK; ^2^​ Emergency Department, Sheffield Teaching Hospitals, Sheffield, UK; ^3^​ Department of Landscape Architecture, University of Sheffield, Sheffield, UK

**Keywords:** public engagement, science capital, marginalized ethnic groups, student-led, impact, science art

## Abstract

There is a wealth of innovation in microbiology outreach events globally, including in the setting where the public engagement is hosted. Previous data indicate an underrepresentation of marginalized ethnic groups attending UK science-based public engagement events. This project engaged our student cohort, encompassing a diverse range of ethnic groups, to create an integrated art and science event within an existing series of adult education evenings. The study’s objectives were to increase the proportion of visitors from marginalized ethnic groups and to gain a greater understanding of the impact of the event on the visitors’ reported science capital. The participants’ demographics, links to our students and University, and detailed impact on participants’ science capital of the event were determined through analysis of exit questionnaires. There was an increase in the proportion of marginalized ethnic group visitors compared to similar previous events. A higher proportion of visitors from marginalized ethnic groups had links with our students and University compared to white/white British visitors. Elements of the exit questionnaire were mapped to the science capital framework and participants’ science capital was determined. Both ethnically marginalized participants and white/white British visitors showed an increase in science capital, specifically dimensions of science-related social capital and science-related cultural capital, after the event. In conclusion, our study suggests that a student-led blended art and science public engagement can increase the ethnic diversity of those attending and can contribute towards creating more inclusive public engagement events.

## Data Summary

The data presented in this study may be available on request from the corresponding author. The data are not publicly available due to ethical restrictions.

## Introduction

The UK National Co-ordinating Centre for Public Engagement defines public engagement as ‘the myriad of ways in which the activity and benefits of higher education and research can be shared with the public’. Engagement is by definition a two-way process, involving interaction and listening, with the goal of generating mutual benefit. The increasing narrative to take public engagement out into individual communities has led to the establishment of creative and innovative events with reported success in reaching audiences who typically would not engage with science activities [1–4].

The public’s engagement in science, trust in scientists and trust in scientists’ work has individual and societal benefits [5, 6]. The public engaging with science allows individuals to make informed decisions around their own lives, and more widely this decision-making impacts society as a whole. When sections of the community do not trust scientists there is often a negative impact for that group of society. For example, vaccine hesitancy amongst subgroups within the population, including ethnic minority communities during the Covid-19 pandemic [7], is a significant health threat globally. Whilst the science–societal relationship is complex, public engagement events give science a platform to create a dialogue between scientists and the public; however, we must ensure that events are accessible to all.

Public engagement strategies aspire to engage with groups that fully represent society [8, 9]. Race and ethnicity-based inaccessibility and misrepresentation is reported to be an important barrier in engagement with science events [10]. Communities that scientists find difficult to engage are consistently underrepresented in the visitor demographics at such events, including marginalized ethnic groups [2, 11]. This highlights the importance of culturally appropriate platforms. Inclusive science communication can help societal progress by addressing the inequitable distribution of and engagement in science [8]. Subsequently, the development of successful and inclusive public engagement models could allow practitioners to rethink approaches to public engagement activities.

### A sense of belonging

People with a strong science identity, such as those who identify themselves as a ‘science person’, are more likely to feel a sense of belonging in and/or amongst science [12, 13]. A person’s sense of belonging is key to their likelihood to seek out, stay and succeed in a space. This holds for scientific communities, where people’s perception of themselves as valued community members affects their attainment and retention [14, 15]. People from underrepresented groups tend to feel a lower sense of belonging in science [13, 16, 17] and report increased accessibility barriers leading to social exclusion from engagement with science public engagement events [10]. Interventions which increase the sense of belonging in a member of an underrepresented or disadvantaged group can increase engagement and attainment in science [12, 18, 19].

Role models can play key roles in establishing a sense of belonging in members of underrepresented groups [15]. Exposure to similar role models in science helps members of underrepresented groups overcome stereotypes that science is not ‘for them’, and thus helps develop their science identity [20–22]. While role models can be a factor in a person’s sense of belonging, this effect varies depending on the similarity of the role model, with role models perceived as relevant and compatible with a person’s identity more likely to have a positive impact on that person [22–24].

### Aims

Building on our previous work undertaking public engagement of science in a non-science space, this study aims to evaluate the impact of engaging a diverse body of student organizers and presenters in a blended science and art event hosted in a public gallery on the impact of the resulting audience demographic. Through evaluation of exit questionnaires, we wanted to gain a greater understanding of the impact of attending the event across different groups of visitors through a science capital lens.

**Research question 1:** Can a student-led public engagement event attract an ethnically diverse audience which is representative of the local regional demographic?

**Research question 2:** Does the perceived learning gain and immediate reported impact on science capital differ between visitors from marginalized ethnic groups and white/white British visitors?

### Science capital framework

How well an individual feels connected with science and their feelings towards science can be explored through the science capital framework. Derived from the social theory of capital, science capital is described as the ‘science-related resources’ to which an individual has access [25]. Dimensions of science capital include science-related cultural capital, an individual’s engagement and participation in science, and science-related social capital, such as who you know who works in science. With positive attitudes towards science being related to higher levels of science capital, using the lens of science capital can help to explain variable rates of participation in science across society including ethnically marginalized and socioeconomically disadvantaged communities [26].

There is a drive to build and enhance science capital amongst the public to allow continued societal support for science and widened engagement across the breadth of society [27]. Previously we have reported that both community [2] and university-hosted [28] events can increase knowledge and elements of science capital amongst participants, with significantly higher reported knowledge gain in visitors from low progression to higher education postcode areas [28]. These findings are mirrored within the literature, with several studies showing that through engaging with informal science activities many participants report an increase in their science capital and more positive attitudes towards science [29, 30]. Unfortunately, we, and much of the science community, are still failing to attract audiences to events which are ethnically diverse and representative of society and thus those communities we find harder to reach often have lower science capital [2, 11, 28, 31]. Science capital will be used as a framework to address the aims of this study.

## Methods

### Event

**Fig. 1.**
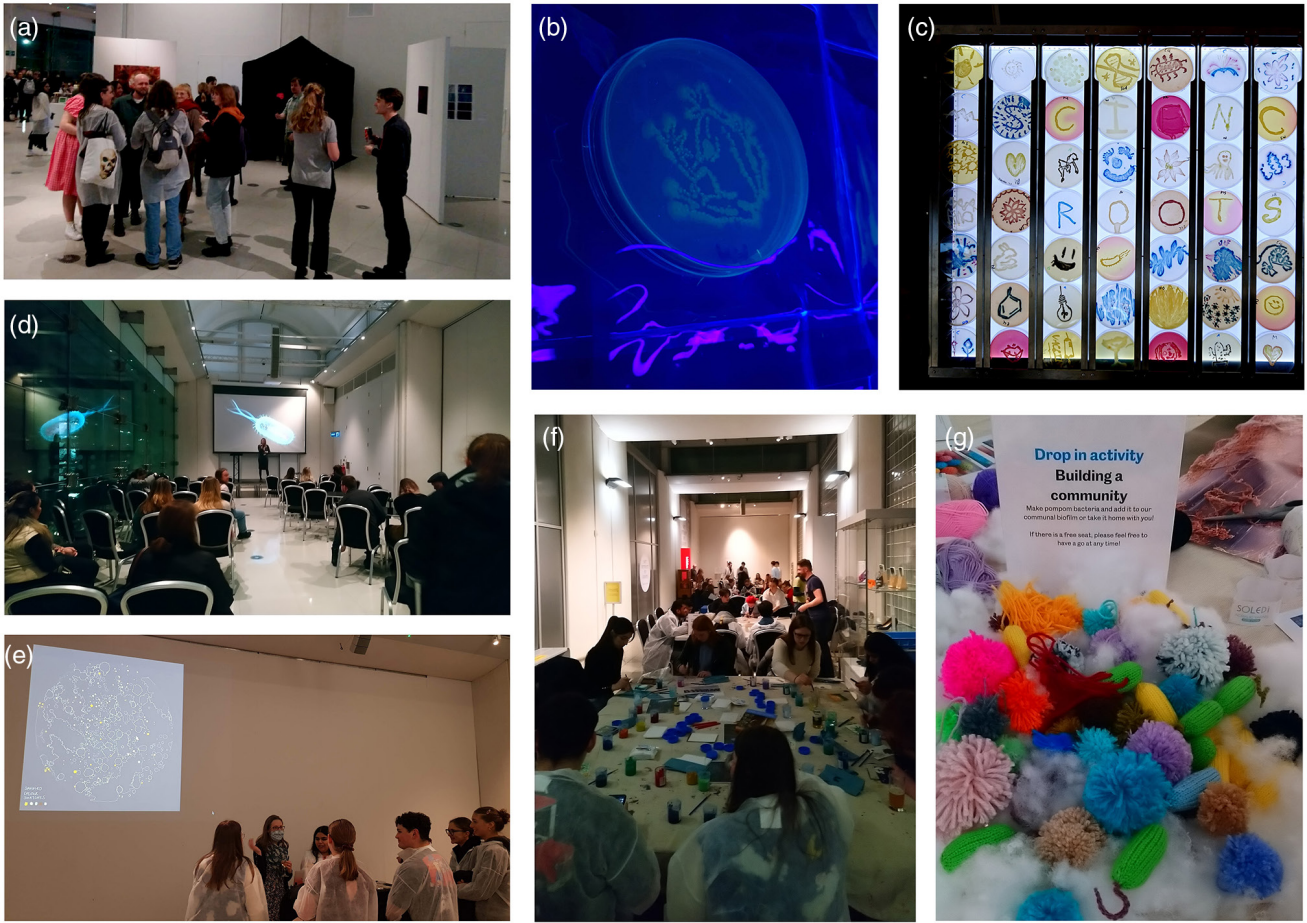
Elements of the Art of Science event. (**a**) Science image exhibition area with visitors discussing research topics with doctoral students, (**b**) student-designed fluorescent bacteria agar art housed in the blackout tent, (**c**) ‘Science Roots’ light box exhibition where undergraduate students created agar art with the theme of what inspires them to study science, (**d**) mini-lecture series which ran throughout the evening, (**e**) the artist in residence created a visual projection on the main wall of the exhibition after shadowing student researchers undertaking microbiology research, (**f**) multiple hands-on creative art activities based around microbiology research and (**g**) visitor-created piece of individually crafted bacteria forming a biofilm.

The ‘Art of Science’ event was hosted at the Millenium Galleries in Sheffield City Centre. The event was a collaboration between Sheffield Hallam University and Sheffield Museums Trust. As with previous collaborative projects [2] the Art of Science was a multifaceted, informal, one-off event, after the normal opening times of the museum and gallery space for those 16 years old and older. The event was predominately advertised by Sheffield Museums Trust, through their newsletter and social media platforms. As with previous events, the authors invited staff and students within the Department of Biosciences and Chemistry through email via the virtual learning platform. The science and art event could be viewed as three sections, the ‘art gallery’, hands-on art of science activities and a mini-lecture series (Fig. 1, Table 1). The Millennium Galleries provided exclusive tours of the exhibits and additional hands-on experiences including print making and felt crafts inspired by the natural history collections of the museum. Undergraduate and postgraduate students were invited to the event both as volunteers and as visitors.

**Table 1.** Elements and activities at the Art of Science event

Element/activity	Overview	Resources	Student-led input
**Art Gallery**			
Art of Science Exhibition (Fig. 1a)	Single, striking images of research were displayed in an art gallery style	High-quality printed images and display boards	PhD students presented representative images from their work and held conversations with the public on their research topics
‘Science Roots’ Agar Art (Fig. 1b)	Agar art around the theme of ‘science roots’ was displayed in a backlit box	Nutrient agar, MacConkey agar, Mannitol salt agar, UTI brilliance agar. *Staphylococcus aureus* SH1000, *Staphylococcus epidermidis* ATCC 14990, *Pseudomonas aeruginosa* PAO1 and *Escherichia coli* MG1655	Final-year undergraduate students and MSc students from the Department of Biosciences and Chemistry were given the opportunity to make agar art under the theme of ‘scientific roots’
Black-out Tent Art (Fig. 1c)	Luminescent agar art was displayed in a black-out tent with UV light sources	Marine agar and *Vibrio harveyi* BAA-1116	Students helped produce luminescent agar art to display in the black-out exhibition
The Artist in Residence Exhibition (Fig. 1e)	A series of projected images and short animations inspired by soil microbiology research	Audio-visual equipment for projection. Digital drawings using iPad. Watercolour paper, acrylic inks, Indian ink, pencils, soluble graphite	Prior to the event, the artist had visited MChem and MSci students in the microbiology research laboratories to find out more about and gain hands-on experience of the project before creating the exhibition pieces
**Hands-on activities**			
Building a Community (Fig. 1g)	Visitors helped mature the microcolony of knitted and crocheted bacteria into a woolly, mature, polymicrobial biofilm with pom poms	Wool, scissors and cardboard	Students volunteered to help run the stand and discuss the concept of microbial biofilms with event attendees
Reinventing Life Drawing	Participants swabbed their own oral microbiome and then drew onto agar plates	Dry, sterile swabs and nutrient agar plates (incubated overnight at 37 °C after the event)	Students volunteered to help run the stand and used the activity to engage members of the public on discussions around the human microbiome. They also took photos of and shared the art on social media for visitors to see after the event [38]
Pastel Pathogens	Visitors observed a range of pathogens under the microscope, creating a pastel picture in response	Light microscopes and Gram-stained bacterial slides. Pastels and pastel paper	Students volunteered to help run the stand and discussed concepts in microbial pathogenesis and infection control with event attendees
Soil Microbiome Project	Participants explored the impact of pollution on soil through colour-dots paintings of soil components	Acrylic paint, small brushes, acrylics, paper	Students volunteered to help run the stand and discuss the topic of soil pollution and environmental microbial communities with attendees
**Mini-lecture series** (Fig. 1d)			
Mini-lectures	The event offered a mini-lecture series of talks from academic staff and PhD students from the Department of Bioscience and Chemistry focused on their areas of research interest	AV equipment	PhD students were given the opportunity to present their research projects

### Data collection

Exit point feedback from visitors was collected using a modified version of our previously designed mixed-methods questionnaire [2]. The paper questionnaire (Supplementary materials, available in the online version of this article) was designed to be quick to complete to maximize completion by participants. It consisted of a combination of simple profiling tick boxes, Likert-style responses and free text comment boxes. After the event, the questionnaire data were manually transferred into Excel for analysis.

### Data analysis: visitor demographics, enjoyment and perceived learning

Open coding was used to code free text responses of the question ‘Tell us something from your visit that you have found particularly interesting’, followed by thematic analysis and categorization into themes [32].

Visitors self-identified ethnicity within the categories of Asian/Asian British, black/black British, mixed ethnicity, other and white/white British. These categories of ethnicity were taken from the Sheffield 2011 Census [33] to allow comparison of the ethnicity of visitors with the Sheffield region and previous collaborative events between the research team and Sheffield Museums Trust [2]. Ethnically marginalized groups is defined within this piece of work as participants within black/black British, Asian/Asian British, mixed ethnicity and other categories.

As a measure of the perceived learning by visitors, participants were asked to rate their pre- and post-visit knowledge of the six key microbiology event topics: microbes in the body, microbes that cause disease, microbes in the soil, biofilms, antibiotics and DNA. Scores were subsequently added to create an overall individual perceived learning score for each participant. Differences between groups was determined by a Wilcoxon rank sum test, and statistical analysis was performed in R v4.1.

### Data analysis: science capital

Participants’ existing and expected future engagement with science were used as a measure of event impact on science capital. Nine Likert-style engagement questions were designed to cover key dimensions of science-related capital, namely scientific literacy, science-related attitudes, values and dispositions, science media consumption, participation in informal science events, and talking about science in everyday life [31]. Knowledge about the transferability of science was not included in this study as it focuses on the knowledge of science qualifications linking to jobs, which was not touched upon in the event. In addition, participants were asked about their highest level of science qualification and whether they and/or someone close to them worked in the science industry as additional measures of science-related social capital [25] (Table 2).

**Table 2.** Framework for science capital data collection and analysis Individual elements of science capital were mapped to question(s) on the exit questionnaire and each element analysis to give a score from 0 to 1. Science-related social and cultural capital scores were determined from the respective elements and given a score from 0 to 1 and finally overall science capital score was determined from the science-related social and cultural capital score and put on a 0–1 scale.

	Question(s)	Analysis. N.B.: no. is initial score allocated to each question response
1 Science capital	n/a	1.1 and 1.2 scores
1.1 Science-related social capital	n/a	1.1.1–1.1.3 scores
1.1.1 Family science skills, knowledge and qualifications	(a) ‘Do you work in science?’ (b) ‘What is your highest qualification?’	1 No, 5 Yes 1 GSCE/O level, 2 A level or equivalent, 3 BSc, 4 Masters, 5 PhD
1.1.2 Knowing people in science-related roles	‘Do any of your family or friends work in science?’	1 No, 5 Yes
1.1.3 Talking about science in everyday life	‘I regularly discuss science with family and friends’	Likert scale of 1 strongly disagree to 5 strongly agree: before and after event
1.2 Science-related cultural capital	n/a	1.2.1–1.2.5 scores
1.2.1 Scientific literacy	(a) ‘How much do you know about the following, before visiting and after visiting … Microbes in the body, Biofilms, DNA, Microbes that cause disease, Microbes in the soils, antibiotic resistance?’ (b) ‘I feel confident talking with others about science’	Likert scale of 1 nothing to 5 a lot: before and after event for each topic Likert scale of 1 strongly disagree to 5 strongly agree: before and after event
1.2.2 Science-related attitudes, values and dispositions	(a) ‘Science is useful to me in my daily life’ (b) ‘Science is important in society’ (c) ‘I believe science is everywhere’ (d) ‘Scientists do valuable work’	(a–d) Likert scale of 1 nothing to 5 a lot: before and after event for each question
1.2.3 Knowledge about the transferability of science	Not included in questionnaire	n/a
1.2.4 Science media consumption	‘I actively engage with/look for books/magazines/TV or internet content about science’	Likert scale of 1 nothing to 5 a lot: before and after event for each question
1.2.5 Participation in out-of-school science learning contexts	‘I regularly (at least twice a year) visit science museums, festivals and/or science-focused events’	Likert scale of 1 nothing to 5 a lot: before and after event for each question

Scores of each question on the questionnaire were scaled to a value between 0 and 1. Likert scale responses were scaled to a range of 0.2–1; for example, a Likert scale score of 4 translated into 0.8. ‘Yes’ or ‘No’ responses were given values of 1 or 0 respectively. The mean of the scaled scores was used where multiple questions relate to a single dimension. The score of cultural and social capital was an average of the dimensions within them. Scores of each capital and dimension were used to create a heat map, the colours of which were used to colour the hierarchy graph. Dimensions were compared before and after the event by Wilcoxon signed rank tests and between ethnicity groups at each time point by Mann–Whitney tests. Data analysis was performed in Prism v8.1.1 (GraphPad Software).

## Results

To determine the impact of the Art of Science event on participants’ science capital, as well as the uptake and impact of visitors from marginalized ethnic groups, exit questionnaires were undertaken. The event had 282 visitors with 123 completing an exit questionnaire, and thus a 44 % uptake.

An individual’s learning is positively linked to their engagement and enjoyment of a topic or activity [34]. The question ‘Tell us something from your visit that you have found particularly interesting’ was thematically analysed to determine aspects of the event that participants found engaging (Table 3).

**Table 3.** Qualitative analysis themes of participants’ interest Answers to ‘Tell us something from your visit that you have found particularly interesting’ were blinded, coded into each category and enumerated. Example comments are given for each theme (*n*=104).

Themes	Example	No. of responses
Specific scientific/factual learning points	‘Bioluminescence’, ‘background microbes’, ‘antibiotic resistance’	45
Talks/lectures	‘Oral cavity’, ‘bone structure’	7
Opportunity to learn something new	*‘*Excellent science communication to a non-scientist’, ‘translating science’	5
Opportunity to be creative/science inspiring art	*‘*Amazing shapes and patterns of the micro world’, ‘thrush looks like grapes’	25
Positive overall experience	*‘*Love the lady studying mine water’, ‘passion from the presenters’	7
Interactive activities	*‘*Using a microscope’, ‘handling fossils’	11

The responses identify specific scientific and factual learning as the most interesting element of the Art of Science event followed by the opportunity to be creative and artistic. There was no difference in the theme of response based on participants’ ethnicity (data not shown).

### Student involvement putatively increased the number of visitors from marginalized ethnic groups

An aim of the project was to increase the proportion of visitors from marginalized ethnic groups at the event. The ethnicity of participants of the Art of Science was compared to previous collaborative events with Sheffield Museums Trust and the Sheffield region (Table 4).

**Table 4.** Comparison of participant ethnicity (%) at the Art of Science event compared to previous collaborative events in the Sheffield region Art of Science (*n*=123), The Horror Within and The Science of Science Fiction with Sheffield Museums Trust [2] and Sheffield Census [33]. Note where percentages do not equal 100 % for an event, the absent participants chose to not disclose their ethnicity.

Ethnicity	Art of Science (2022) (*n*=123)	The Horror Within (2017) (*n*=51)	The Science of Science Fiction (2018) (*n*=51)	Sheffield Census (2011) (*n*=552,698)
Asian/Asian British	13.1	4.1	5.8	8.0
Black/Black British	2.5	0.0	0.0	3.6
Mixed	3.3	2.0	5.8	2.4
Other	1.6	0.0	0.0	2.2
White/White British	77.9	93.9	88.5	83.7

The demographic of visitors at the Art of Science event was markedly different compared to previous blended art and science evenings. The Art of Science event had an increase in the proportion of all marginalized ethnic groups apart from mixed ethnic when compared to the Science of Science Fiction event. The most marked increase was the increase in Asian/Asian British participants, increasing to 13.1 % compared to 4.1% and 5.8 % for the previous events. There was also an increased proportion of Asian/Asian British and mixed ethnicity participants compared to the Sheffield region, although black/black British and other ethnicities were underrepresented at the Art of Science event compared to the Sheffield region.

To determine if the increase in the proportion of participants at the Art of Science event from marginalized ethnic groups was due to the social-capital impact of increased student-led participation, the ‘How did you hear about the event?’ question was analysed (Table 5).

**Table 5.** Comparison of how people heard about the Art of Science event Due to the sample size, all marginalized ethnic participants were analysed together (all responses *n*=123, marginalized ethnic participant responses *n*=26, white/white British *n*=95).

	Museums Sheffield Trust website/poster	Sheffield Hallam website/poster	Social media	I know someone involved in the event	Friend/family	Other
Total	12 (10 %)	10 (8 %)	37 (30 %)	23 (19 %)	30 (24 %)	11 (9 %)
Ethnically marginalized groups	3 (12 %)	5 (19 %)	7 (27 %)	6 (23 %)	5 (19 %)	0
White/white British	9 (9 %)	4 (4 %)	29 (31 %)	17 (18 %)	25 (27 %)	11 (11 %)

Participants from marginalized ethnic groups were slightly less likely to hear through social media than white/white British participants (27 and 31% respectively), and slightly more likely to attend the event through knowing someone involved (23 and 18% respectively). Participants from marginalized ethnic groups were much more likely to hear from a Sheffield Hallam University website or poster than white/white British participants (19 and 4% respectively).

### Impact of attending the event was seen across all visitors, with differences observed between white/white british and marginalized ethnic group participants

**Fig. 2.**
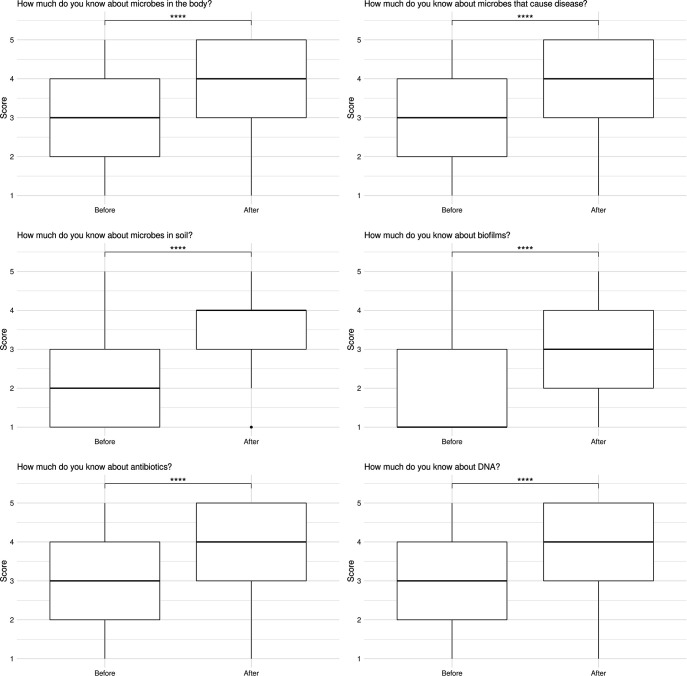
Perceived knowledge before and after of different areas. The amount of perceived knowledge participants gained during the Art of Science event in the six science content areas was ranked from 1 (nothing) to 5 (a lot). Data are shown as median values at the centre of the plot, first and third quartiles complete the plot and the whiskers represent 1.5×IQR from quartiles. Outlying points are represented as individual points (*n*=123). ****P≤0.0001 in a Wilcox signed rank test.

The main scientific content for the Art of Science event was broadly categorized into six themes: microbes in the body, biofilms, DNA, microbes that cause disease, microbes in the soils and antibiotic resistance. To determine perceived learning at the event, participants were asked ‘How much do you know about the following?’ for each theme, before and after the event on a scale of 1 (nothing) to 5 (a lot) (Fig. 2).

Exit questionnaire analysis showed an increase in perceived learning by participants in all main themes of the Art of Science event. There was no difference in the perceived learning of participants from marginalized ethnic groups compared to their white/white British counterparts (data not shown). Due to the nature of data collection, we were unable to determine if participants perceived learning increases were accurate.

**Fig. 3.**
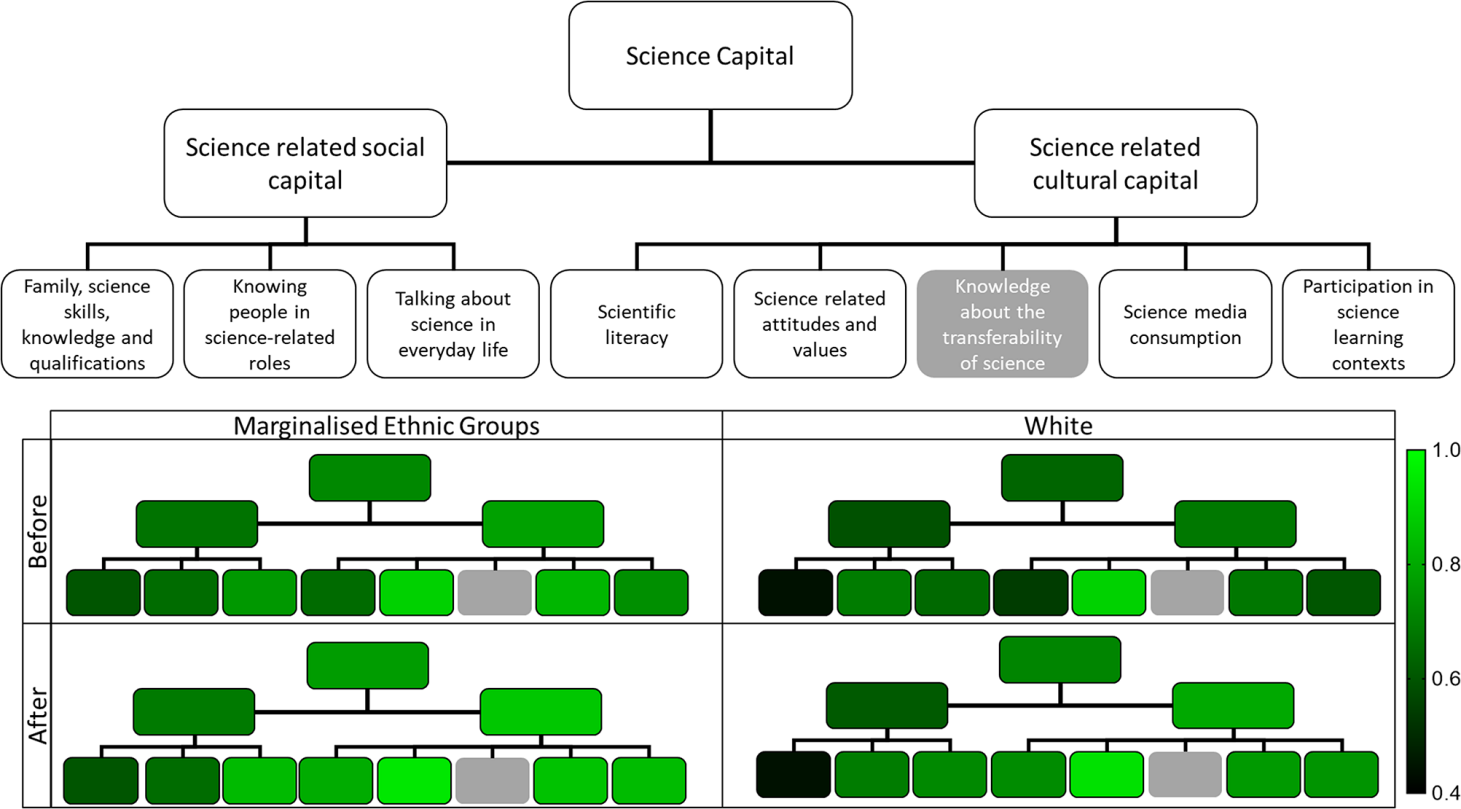
Impact of attendance at the Art of Science event on science capital and related dimensions. The mean score of each dimension was grouped by ethnicity and before/after attendance at the event. The higher level dimensions were an average of sub-dimensions. The range of results was between 0.44 and 0.94, indicated by a gradient scale from black to green. Hierarchy plots mimic the layout of the top plot of science capital dimensions (*n*=123).

Perceived learning forms part of the science capital framework. Using the framework outlined in Table 1, participants’ exit questionnaires were analysed to determine differences between marginalized ethnic participants and white/white British participants’ science capital. The framework allows investigation of two elements of science capital: first, participants pre-existing science capital and second, the impact of the event on participants’ science capital (Fig. 3).

### Participants’ pre-existing science capital

No difference in pre-existing overall science capital and science-related social capital was observed between marginalized ethnic participants and white/white British participants. Participants from marginalized ethnic groups had a higher pre-existing science-related cultural capital score than those from white/white British backgrounds (*P*<0.05, Mann–Whitney test). Within the individual elements of science-related social capital, participants from marginalized ethnic groups had a higher score in ‘family, science skills, knowledge and qualifications’ than those from white/white British backgrounds (*P*<0.05, Mann–Whitney test). There was no statistically significant difference in the remaining individual elements. Within the individual elements of science-related cultural capital, participants from marginalized ethnic groups had a higher score in ‘scientific literacy’, ‘science media consumption’ and ‘participation in science learning contexts’ than those from white/white British backgrounds (*P*<0.05, Mann–Whitney test). There was no statistically significant difference in ‘science-related attitudes and values’ and it is worth noting that this element scored the highest across the framework analysis.

### Impact of the event on participants’ science capital

Participants from both marginalized ethnic backgrounds and white/white British backgrounds reported an increase in their overall science capital after the event. They also reported an increase in both its components, science-related social capital and science-related cultural capital (*P*<0.05, Wilcoxon matched pairs signed rank test). Within the individual elements of science-related social capital, both groups of participants had a higher score in ‘talking about science in everyday life’ after the event (*P*<0.05, Wilcoxon matched pairs signed rank test). There was no statistically significant difference in the remaining individual elements between elements based on relationships. Within the individual elements of science-related cultural capital, both groups of participants had an increase in ‘scientific literacy’ and ‘participation in science learning contexts’ (*P*<0.01, Wilcoxon matched pairs signed rank test). Finally, white/white British participants reported an increase in ‘science-related attitudes, values and dispositions’ and ‘science-media consumption’ (*P*<0.01, Wilcoxon matched pairs signed rank test) due to the event, whereas no difference was seen for marginalized ethnic groups.

## Discussion

Drawing on the previous success of blended arts and science events, a science public engagement event was hosted in a non-science space. Our previous events, also hosted in a museum setting, engaged a ‘non-science’ audience although participants from marginalized ethnic groups were underrepresented compared to the local population [2]; this was consistent with the UK national picture for museum attendees [35, 36]. This study employed a university student-led approach to the Art of Science event, aiming to increase the ethnic diversity of those attending. Our study also explored event impact on visitors from marginalized ethnic communities and white/white British communities through exit questionnaires and qualitative data analysis.

With continued underrepresentation of visitors from marginalized ethnic groups at science public engagement events, inequality in science public engagement remains [8]. Key barriers to marginalized and minoritized individuals and communities are reported as a lack of a sense of belonging, accessible role models and low levels of existing science capital [12, 15, 26]. The student body in the Department of Biosciences and Chemistry at Sheffield Hallam University has a higher representation of individuals from marginalized ethnic groups (~30 %) than the Sheffield City Region population (16.3%) [2]. Our approach was to engage these students in the organization, preparation and delivery to increase the ethnic diversity of those attending the Art of Science event. Briefly, this approach draws upon existing literature around relatable role models increasing the sense of belonging and engagement in science amongst minoritized and marginalized individuals and groups [12, 15, 22].

Exit questionnaires were used to capture the demographics of participants and the immediate impact of the event. Previous similar events undertaken by the research team have echoed the national picture, which sees white individuals more likely to visit museums and science spaces than those from marginalized ethnic groups [2, 35, 37]. The author’s previous events (Horror Within and the Science of Science Fiction: [2]) were both hosted in the same gallery space as the Art of Science, were both one off, 16+ events and both blended art and sciences. The Art of Science event observed an increase in the proportion of visitors from marginalized ethnic groups (20.5 %) in comparison to our previous bended art and science events (6.1 % in 2017 and 11.6 % in 2018) [2]. This was also above that of the Sheffield City region at 16.3 % for marginalized ethnic citizens [33]. The methods to generate these data was the same for all three events, a paper exit questionnaire with the same categories for ethnicity self-selection which was based on the categories within the 2011 National census. The ethnic diversity of day visitors to the Millenium Galleries in 2023 was similar to the overall Sheffield region, but data for the more comparable evening events were not collected (unpublished data courtesy of Sheffield Museums Trust).

Overall, social media led as the most common way visitors had heard about the event. However, participants from marginalized ethnic groups were more likely than white/white British participants to have heard about the event through someone involved or via Sheffield Hallam advertising. The increase in ethnic diversity was not equivalent across all ethnic groups, with Asian/Asian British having the higher representation at the event compared to the Sheffield Census. Interestingly, there is a higher proportion of Asian/Asian British students within our department than black/black British. Whether the increase in Asian/Asian British visitors is a direct result of this can only be speculated upon. Correlation does not equal causation, and our research of a university student-led art and science event, with a diverse student demographic and the subsequent increase in the ethnic diversity of attendees, is an example of such. The comparison with previous events [2] and the data around how different groups of visitors heard about the event adds circumstantial evidence to a causal link. It is tempting to speculate that students involved in the event, either in creating agar or as a volunteer, encouraged friends and family to attend, and those from minoritized ethnic groups with no direct link to the event team would perhaps feel more welcome as they could see their peers involved and thus the space was more accessible. To strengthen these hypothesis, further events could include short exit interviews with visitors to further explore their reasons for attending the event.

Others have reported that there can be barriers to engagement within event exhibits for minority ethnic visitors, for example due to language, which ultimately lead to the feeling of not belonging and to unease [10]. There was no difference observed at this event in the reported knowledge gain or interests between the Art of Science minoritized ethnic and white/white British visitors. It is acknowledged that our minoritized ethnic group visitors had higher existing science education, which potentially impacted on the responses to these questions. However, working with our diverse student organizers to prepare and deliver the event could have contributed towards making an inclusive accessible event and minimized any implicit biases in design that may be hindering rather than aiding in promoting inclusivity.

An individual’s relationship with and attitude towards science is influenced by their science capital [25]. Understanding levels of science capital amongst different groups of the population can help explain social inequalities in science participation [25, 26]. Through participant exit questionnaire responses we found no difference in the overall existing (pre-event) science capital scores between marginalized ethnic groups and white/white British visitors. Further analysis of the dimensions of science capital explored in the questionnaire did identify higher cultural capital scores (across all elements) in marginalized ethnic visitors when compared to white/white British visitors. Visitors from marginalized ethnic groups also reported knowing more people working in science and holding higher level science qualifications than white/white British visitors. It is encouraging that our study suggests that students, as a diverse organization and presenting body, can increase ethnic diversity at a science-based event, although the resulting participants from marginalized ethnic groups have a higher existing level of some elements of science capital before attending than white/white British visitors. We have previously shown that hosting a blended science and art event in a non-science space can attract and engage visitors who typically do not engage with science [2] and whilst our current study suggests an approach which can also increase ethnic diversity, these visitors are already more engaged in science through their existing reported science capital. Dawson [31] argues that science communication is not open to everyone due to social advantage and structural inequalities, meaning that events remain invisible to some groups in society. Our study suggests that whilst involving diverse multiple voices in planning and delivery through recruitment of our student body could broaden the reach of science public engagement events in non-science spaces such as museums, additional barriers are preventing societal groups of minority ethnic citizens with low levels of existing science engagement from participating.

Collective science capital scores for participants of both marginalized and white ethnic backgrounds were reported as being increased after visiting the event. With participants reporting that they were more likely to talk about science in everyday life and participate in future science events, the Art of Science event successfully increased accessibility of science to all visitors. This equal impact gain across both white/white British and marginalized ethnic group participants, together with the knowledge gain and interest discussed earlier, suggests that our student-led event model is a move in the right direction of inclusive science public engagement.

## Conclusion

A student-led Art of Science event was evaluated via exit questionnaires. Ethnic diversity was increased amongst visitors compared to previous events by the group as well as the Sheffield region. A sizeable minority of participants, higher in ethnically marginalized groups, at the event reported attending due someone they knew being involved through the university or through a university poster or website. Thus, it is tempting to speculate that the increase in ethnicity was in part due to an increase in the ethnic diversity of those involved in planning and organization.

A science capital framework was used to gain a better understanding of the impact of the event on participants. Several pre-existing elements of science capital were higher in participants from marginalized ethnic groups than white/white British visitors. Overall, reported science capital was increased in visitors irrespective of ethnicity and this increase was seen in discrete elements of science capital.

This student-led blended art and science event contributes towards creating a more inclusive science public engagement approach. However, complex barriers are still in place surrounding participants from ethnically marginalized groups attending public engagement events, and a greater understanding of the rich diversity within ethnically marginalized groups will allow future events to engage more fully with diverse communities.

## Supplementary Data

Supplementary material 1
