# Supplementary material for: University student-led public engagement event: increasing audience diversity and impact in a non-science space
Source: Access Microbiol. 2023 Aug 29;5(8):acmi000534.v3. doi: 10.1099/acmi.0.000534.v3 (PMC10484318; doi:10.1099/acmi.0.000534.v3)
Supplement: Supplementary material 1 [file acmi-5-534.v3-s001.pdf]

Art in Science 2022 – please leave your feedback

How much do you know about the following?

	**Before visiting**					**After visiting**				
	Nothing		A little		A lot	Nothing		A little		A lot
Microbes in the body	1	2	3	4	5	1	2	3	4	5
Biofilms	1	2	3	4	5	1	2	3	4	5
DNA	1	2	3	4	5	1	2	3	4	5
Microbes that cause disease	1	2	3	4	5	1	2	3	4	5
Microbes in the soil	1	2	3	4	5	1	2	3	4	5
Antibiotic resistance	1	2	3	4	5	1	2	3	4	5

Tell us something from your visit that you have found particularly interesting

To what extent do you agree or disagree with the following statements?

	**Before visiting**					**After visiting**				
	Strongly Disagree				Strongly Agree	Strongly Disagree				Strongly Agree
I feel confident talking with others about science	1	2	3	4	5	1	2	3	4	5
I regularly discuss science with family and friends	1	2	3	4	5	1	2	3	4	5
Science is useful to me in my daily life	1	2	3	4	5	1	2	3	4	5
Science is important in society	1	2	3	4	5	1	2	3	4	5
I believe science is everywhere	1	2	3	4	5	1	2	3	4	5
I actively engage with/look for books/magazines/TV or internet content about science	1	2	3	4	5	1	2	3	4	5
I regularly (at least twice a year) visit science museums, festivals and/or science-focused events	1	2	3	4	5	1	2	3	4	5
I regularly (at least twice a year) visit art museums, festivals and/or art-focused events	1	2	3	4	5	1	2	3	4	5
Scientists do valuable work	1	2	3	4	5	1	2	3	4	5

Who are you visiting with? Let us know the number of people of each age, gender and ethnicity in your group.

16-18	19-24	25-34	35-44	45-54	55-64	65+	Prefer not to say

Female	Male	Self-described	Prefer not to say

White / White British	Black / Black British	Asian / Asian British	Mixed Ethnicity	Other	Prefer not to say

What is your postcode?

Do you work in science? Yes ☐ No ☐ **Do any of your family or friends work in science?** Yes ☐ No ☐

What is your highest science qualification? GSCE/O level ☐ A level or equivalent ☐ BSc ☐ Masters ☐ PhD ☐

How did you hear about the event?

Museums Sheffield Trust website / poster etc ☐ Sheffield Hallam University website / poster etc ☐

Social media ☐ Some I know is involved in the event ☐ Other:________________________ **Thank You!**

This study aims to determine who is coming to our events and the impact of the event on visitors. By completing and returning the questionnaire you consent to take part in the study. Once the questionnaire is submitted it cannot be withdrawn from the study as it is anonymous. This study has received ethical approval from Sheffield Hallam University [ER10872482]. If you have any questions about the study or questionnaire you can contact the project led Dr Mel Lacey (m.lacey@shu.ac.uk).

You should contact the Data Protection Officer if: 1) you have a query about how your data is used by the University; 2) you would like to report a data security breach (e.g. if you think your personal data has been lost or disclosed inappropriately); 3) you would like to complain about how the University has used your personal data DPO@shu.ac.uk. You should contact the Head of Research Ethics (Dr Mayur Ranchordas) if you have concerns with how the research was undertaken or how you were treated ethicssupport@shu.ac.uk. Postal address: Sheffield Hallam University, Howard Street, Sheffield S1 1WBT Telephone: 0114 225 5555
